# MicroRNA-425 and microRNA-155 cooperatively regulate atrial natriuretic peptide expression and cGMP production

**DOI:** 10.1371/journal.pone.0196697

**Published:** 2018-04-26

**Authors:** Sara Vandenwijngaert, Clara D. Ledsky, Obiajulu Agha, Connie Wu, Dongjian Hu, Aranya Bagchi, Ibrahim J. Domian, Emmanuel S. Buys, Christopher Newton-Cheh, Donald B. Bloch

**Affiliations:** 1 Department of Anesthesia, Critical Care, and Pain Medicine, Massachusetts General Hospital and Harvard Medical School, Boston, Massachusetts, United States of America; 2 Cardiovascular Research Center, Department of Medicine, Massachusetts General Hospital and Harvard Medical School, Boston, Massachusetts, United States of America; 3 Harvard Stem Cell Institute, Cambridge, Massachusetts, United States of America; 4 Program in Medical and Population Genetics, Broad Institute, Cambridge, Massachusetts, United States of America; 5 Center for Genomic Medicine, Massachusetts General Hospital and Harvard Medical School, Boston, Massachusetts, United States of America; 6 Division of Rheumatology, Allergy and Immunology, Department of Medicine, Massachusetts General Hospital and Harvard Medical School, Boston, Massachusetts, United States of America; Universitat des Saarlandes, GERMANY

## Abstract

**Aims:**

Atrial natriuretic peptide (ANP), secreted primarily by atrial cardiomyocytes, decreases blood pressure by raising cyclic 3’,5’-guanosine monophosphate (cGMP) levels and inducing vasorelaxation, natriuresis, and diuresis. Raising the level of ANP has been shown to be an effective treatment for hypertension. To advance the future development of an anti-microRNA (miR) approach to increasing expression of ANP, we investigated the regulation of *NPPA* expression by two miRs: miR-425 and miR-155. We examined whether miR-425 and miR-155 have an additive effect on the expression and function of ANP.

**Methods and results:**

Human embryonic stem cell-derived cardiomyocytes (hESC-CMs) were transfected with miR-425, miR-155, or a combination of the two miRs. Two days later, *NPPA* expression was measured using real time qPCR. Each of the miRs decreased *NPPA* expression over a wide range of concentrations, with a significant reduction at concentrations as low as 1 nM. The combination of miR-425 and miR-155 reduced *NPPA* expression to a greater extent than either miR-425 or miR-155 alone. An *in vitro* assay was developed to study the potential biological significance of the miR-induced decrease in *NPPA* expression. The cooperative effect of miR-425 and miR-155 on *NPPA* expression was associated with a significant decrease in cGMP levels.

**Conclusions:**

These data demonstrate that miR-425 and miR-155 regulate *NPPA* expression in a cooperative manner. Targeting both miRNAs with anti-miRs (possibly at submaximal concentrations) might prove to be a more effective strategy to modulate ANP levels, and thus blood pressure, than targeting either miRNA alone.

## Introduction

Hypertension is the leading modifiable risk factor for premature death and disability worldwide, and affects more than 1.3 billion individuals [1]. Approximately half of the variability in blood pressure is thought to be genetically determined [2, 3]. Genomic research has spurred tremendous progress in uncovering the genetics of blood pressure regulation and hypertension in humans, and has prompted the development of mechanism-based therapies. One of the pathways regulating blood pressure is the natriuretic peptide system. Atrial natriuretic peptide (ANP) and brain natriuretic peptide (BNP) are hormones that are synthesized and released by cardiomyocytes in response to increased myocardial wall stress. Natriuretic peptides mediate natriuresis, diuresis, and vasodilation by binding to the natriuretic peptide receptor 1 (NPR1), causing increased production of the second messenger cyclic guanosine 3’,5’-monophosphate (cGMP). The dual-acting drug LCZ696 (sacubitril-valsartan), which combines an inhibitor of neprilysin (an enzyme that degrades natriuretic peptides) with an inhibitor of the angiotensin receptor II, has been approved by the US Food and Drug Administration for the treatment of heart failure. In the PARAMETER and other clinical studies [4–6], LCZ696 was shown to lower blood pressure and pulse pressure, rekindling interest in the natriuretic peptide system as a therapeutic target for hypertension and cardiovascular disease.

Genome-wide association studies (GWAS) identified several common single nucleotide polymorphisms (SNPs) in the genes encoding the propeptides of ANP (*NPPA*) and BNP (*NPPB*) that are associated with blood pressure and plasma levels of natriuretic peptides [7, 8]. A genetic variant found to be strongly associated with circulating ANP levels was rs5068, located in the 3’ untranslated region (UTR) of *NPPA* [8]. Individuals carrying the minor allele have higher plasma ANP levels, lower systolic and diastolic blood pressure, and a 15% lower risk of hypertension [8]. The magnitude of the genetic effect of rs5068 on circulating ANP levels was comparable to the change induced by a 20-fold change in dietary sodium intake [9]. Deep sequencing analysis identified rs61764044, a second variant located 123 nucleotides downstream of, and perfectly correlated with, rs5068 (r^2^ = 1); the minor alleles of both variants are always co-inherited. Because of this perfect linkage disequilibrium, rs61764044 is also associated with increased plasma ANP levels, lower blood pressure, and reduced risk of hypertension [10].

Because rs5068 and rs61764044 are always co-inherited, it was not possible to discern the relative contribution of each SNP to the observed effect on plasma ANP levels and blood pressure. Therefore, *in vitro* studies were previously conducted to determine the impact of each SNP on ANP expression. The location of rs5068 and rs61764044 in the *NPPA* 3’UTR raised the possibility that these SNPs interfered with microRNA (miR) binding. MicroRNAs are short noncoding RNAs that mediate post-transcriptional regulation of gene expression by binding to the 3’UTR, resulting in mRNA degradation or translational repression. We have previously shown that rs5068 decreases *NPPA* transcriptional repression by disrupting the binding site of miR-425 in the *NPPA* 3’UTR [9]. Similarly, rs61764044 introduced a G-U “wobble” base pairing within the binding region of miR-155 to the *NPPA* 3’UTR, thereby conferring resistance to miR-155-mediated repression of *NPPA* expression [10]. In human cardiomyocytes, overexpression of either miR-425 or miR-155 induced a significant decrease in *NPPA* expression [10].

Increasing ANP levels, possibly by using miRNA inhibitors (anti-miRs) to interfere with miRNA-mediated repression of *NPPA* expression, is a promising approach to the treatment of hypertension. However, the use of high concentrations of an anti-miR, directed against a single miRNA, might be expected to have unintended (“off-target”) effects on other genes. In contrast, if two miRNAs have an additive effect on gene expression, then targeting both miRNAs (with two different anti-miRs) could allow the use of lower concentrations of each anti-miR, while still achieving the same repressive effect on the target gene. To advance the future development of an anti-miR approach to increasing expression of ANP, we further investigated the joint regulation of *NPPA* expression by miR-425 and miR-155. In particular, we examined whether miR-425 and miR-155 have an additive effect on the expression and function of ANP.

## Materials and methods

### Cell culture

COS7 cells (obtained directly from American Type Culture Collection, CRL-1651) were cultured in Dulbecco’s modified Eagle’s medium supplemented with 10% fetal bovine serum, 2 mM L-glutamine, 200 U/ml penicillin, and 200 μg/ml streptomycin. Cardiomyocytes (CM) were differentiated from human embryonic stem cells (hESC, WA07, NIH Human Embryonic Stem Cell Registry Number 0067, generated by WiCell Research Institute) as previously described [10]. Human ESC-CMs have been well characterized and display functional and structural properties of primary human cardiomyocytes [11, 12], including specifically their expression of *NPPA*, a hallmark of cardiomyocyte-specific expression. Human ESC-CMs express known cardiomyocyte-specific miRNAs and these miRNAs exhibit the expected expression pattern in these cells [13]. Human ESC-CMs were differentiated in RPMI medium supplemented with Gem21 NeuroPlex without insulin, and were maintained in RPMI medium supplemented with Gem21 NeuroPlex (both Gemini Bio Products). Cardiomyocyte differentiation was considered successful when beating clusters of cells were observed and appropriate levels of troponin T (*TNNT2*) mRNA were detected via real-time qPCR.

### Transfection of cardiomyocytes with miRNAs

Cardiomyocytes were harvested between days 19 and 22 of differentiation by washing with PBS and incubation with TrypLE dissociation reagent (Thermo Fisher Scientific) for 12 minutes. Cardiomyocytes were then plated in Matrigel-coated 12-well plates (Corning) at a density of 250,000 cells per well. Four days later, the cells were transfected with double stranded RNAs, chemically engineered to mimic endogenous mature microRNAs (“miRNA mimics”, Thermo Fisher Scientific), using lipofectamine RNAiMax reagent. A random sequence miRNA molecule was used as a negative control for the miRNA mimics. Within each of the experiments, the total amount of transfected miRNA was held constant, either by halving the miRNA concentrations in the combined miRNA conditions or by adding negative control miRNA in the single miRNA conditions. Media was replaced 24 hours post-transfection, and after an additional 24 hours, cardiomyocyte media was collected and cells harvested for RNA isolation.

### Measurement of mRNA levels

RNA was extracted from cardiomyocytes using TRIzol reagent, and reverse transcription was performed using the High Capacity cDNA Reverse Transcription kit according to the manufacturer’s instructions (Thermo Fisher Scientific). Expression of genes encoding *NPPA*, *NPPB*, *TNNT2*, and glyceraldehyde 3-phosphate dehydrogenase (*GAPDH*) was measured by real-time qPCR. The relative cyclic threshold (C_T_) method was used to determine mRNA levels normalized to *GAPDH* mRNA levels [14].

### Measurements of Nt-proANP levels

ProANP (1–98) levels in cardiomyocyte media were assessed using an enzyme-linked immunosorbent assay (ALPCO, Salem, New Hampshire, USA) according to the manufacturer’s instructions.

### Assessment of cGMP levels in NPR1-expressing cells

COS7 cells were plated at 30,000 cells per well in 12-well plates. When the cells reached 60% confluence, X-tremeGENE HP DNA Transfection Reagent (Roche Diagnostics) was used to transfect cells with 0.75 μg of an expression vector (OriGene, pCMV6-AC-GFP vector) containing DNA encoding full-length NPR1 (RefSeq NM_000906) fused to turboGFP (tGFP). Eight hours later, the COS7 media was replaced, and 48 hours after transfection, the transfection efficiency was estimated by visualizing the NPR1-tGFP fusion protein expression via fluorescence microscopy. If the transfection efficiency was at least 40%, the cells were washed with PBS and incubated for 30 minutes with RPMI medium supplemented with Gem21 NeuroPlex without insulin and 0.1 mM phosphodiesterase inhibitor 3-isobutyl-1-methylxanthine (IBMX). The media was then aspirated, and in each well, the cells were incubated for 2 hours with 0.5 ml of media collected from miRNA-treated cardiomyocytes. The media was subsequently removed, and cell lysates were collected and intracellular cGMP levels were measured using a cGMP enzyme immunoassay (Cayman Chemical). The protein concentration of the cell lysates was determined using a bicinchoninic acid assay (Pierce 660nm Protein Assay Reagent, Thermo Fisher Scientific). Cyclic GMP levels were expressed as picomoles of cGMP per milligram of protein and were normalized to cGMP levels in cells incubated with media obtained from cardiomyocytes that had been transfected with negative control miRNA. Standardization of this biological assay was performed by determining the dose-responsiveness of NPR1-tGFP-expressing COS7 cells in terms of change in cGMP levels with increasing concentrations of ANP.

### Statistical analysis

Data are presented as mean ± standard error of the mean. Multiple independent experiments were performed, each with up to 12 technical replicates per condition. Statistical significance of the main effects (e.g. difference in *NPPA* expression compared to negative controls) was assessed using mixed-effects multilevel regression (Stata v14.2) in models including separate experiments as random effects, which is effectively a repeated measures ANOVA. A two-sided *P* value below 0.005 (*P* = 0.05/10 experiments) was considered statistically significant.

## Results

### MiR-425 and miR-155 each decrease *NPPA* expression over a wide range of concentrations

**Fig 1:**
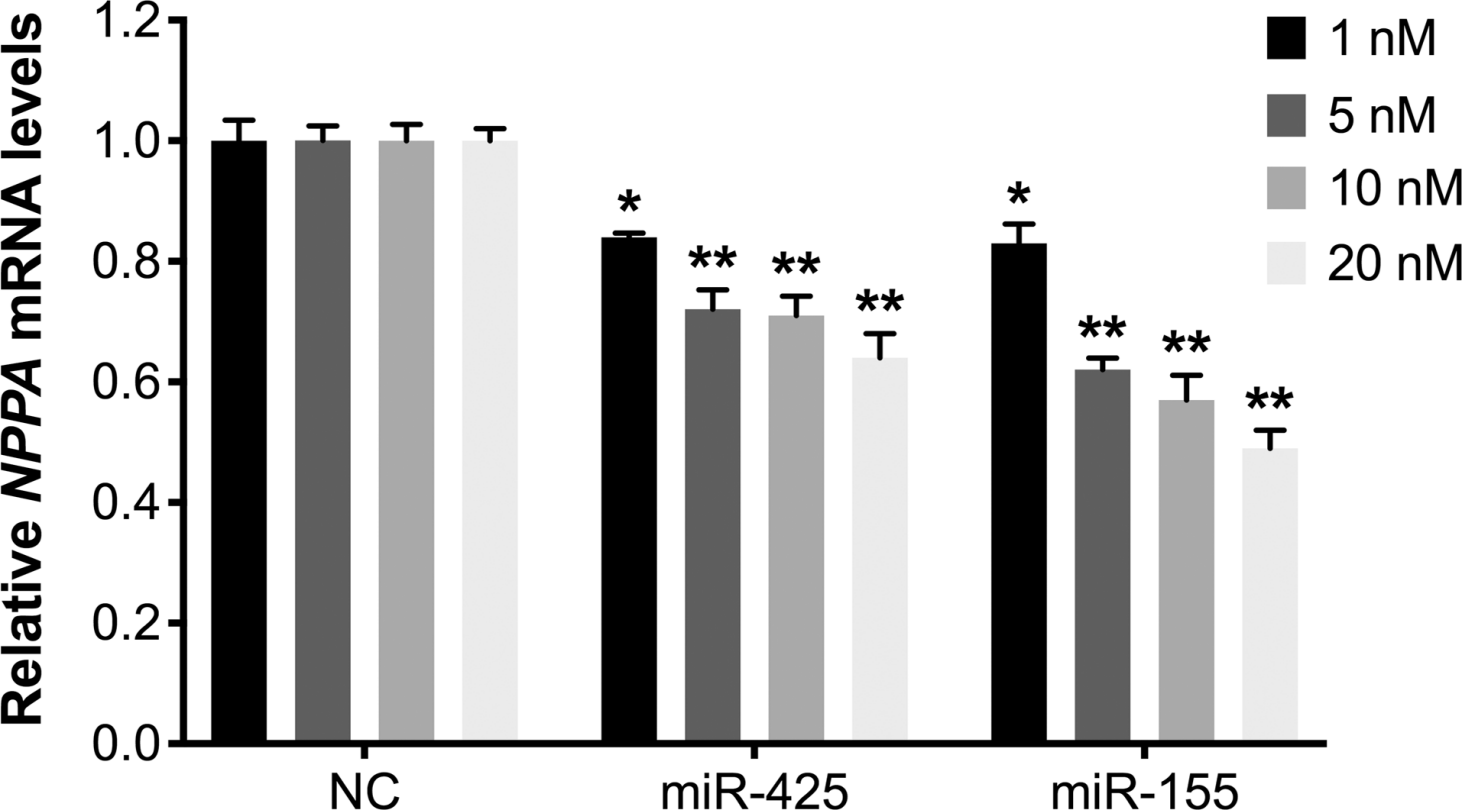
MiR-425 and miR-155 produced dose-dependent decreases in *NPPA* expression over a wide range of concentrations. *NPPA* mRNA levels in human embryonic cell cardiomyocytes (hESC-CMs) transfected with 1 nM, 5 nM, 10 nM, or 20 nM of negative control miRNA (NC), miR-425, or miR-155. Expression is normalized to *GAPDH* expression and shown relative to expression in cells transfected with negative control miRNA. *P<0.01 and **P<1x10^-6^ versus cells transfected with the same concentration of negative control miRNA. N = 4–8 experiments per miRNA concentration (4–12 replicate wells per condition for each experiment).

The objective of this study was to investigate whether miR-425 and miR-155 have an additive effect on the repression of *NPPA* gene expression. A dose-response experiment was performed to identify submaximal concentrations of miR-425 and miR-155 that repress *NPPA* expression, thus providing an opportunity to test the potential, additive repressive effect of the two miRNAs together. In our prior work, we used miRNA mimics at 50 nM, a concentration commonly used in single miRNA studies [10]. In this study, human ESC-CMs were transfected with 1 nM, 5 nM, 10 nM, or 20 nM of miR-425 or miR-155, and *NPPA* mRNA levels were measured 48 hours later. Each of the two miRNAs decreased *NPPA* expression over a wide range of concentrations, with a significant reduction at concentrations as low as 1 nM (Fig 1).

### MiR-425 and miR-155 have an additive repressive effect on *NPPA* expression

**Fig 2:**
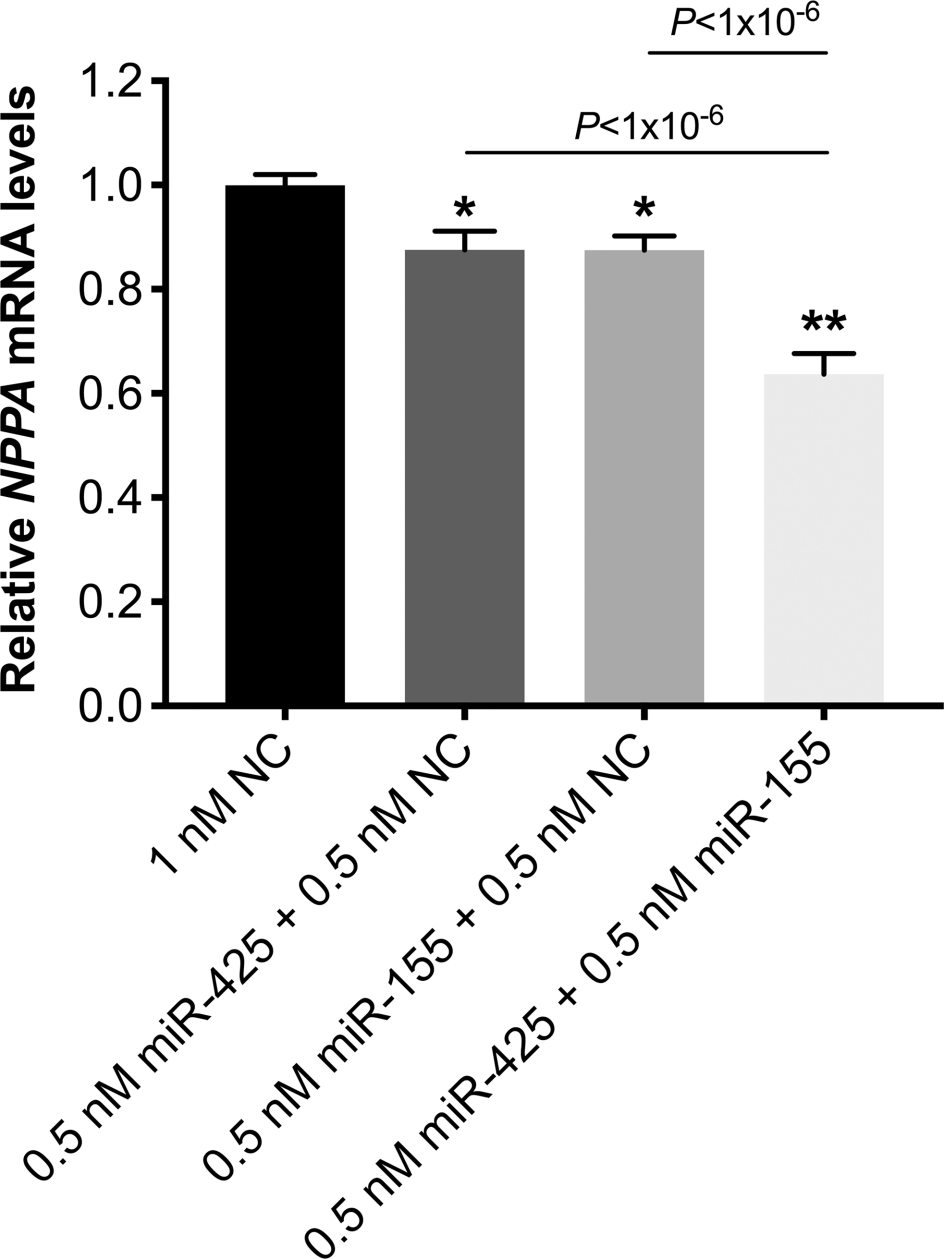
MiR-425 and miR-155 had an additive repressive effect on cardiomyocyte *NPPA* expression. *NPPA* mRNA levels in hESC-CMs that were transfected with either miR-425 or miR-155 alone or with a combination of miR-425 and miR-155. Negative control miRNA (NC) was used as needed to make the total concentration of miRNA constant in each condition. *NPPA* expression is shown relative to that in cells transfected with negative control miRNA. *P<0.01 and **P<1x10^-6^ versus cells transfected with negative control miRNA. N = 3 experiments (6–12 replicate wells per condition).

To determine whether miR-425 and miR-155 exert an additive effect in terms of repressing *NPPA* expression, hESC-CMs were transfected with 0.5 nM of miR-425, 0.5 nM of miR-155, or a combination of 0.5 nM of miR-425 and 0.5 nM of miR-155. In these experiments, negative control miRNA was added so that the total amount of transfected miRNA was constant in each condition. Two days after transfection, the effect of the miRNAs on *NPPA* mRNA levels was evaluated. Compared to the effect of the negative control miRNA, *NPPA* expression was 12% lower in cardiomyocytes transfected with miR-425 alone (0.88±0.04 vs. 1.00±0.02, *P* = 0.001), 13% lower when transfected with miR-155 alone (0.87±0.04 vs. 1.00±0.02, *P* = 0.0004), and 36% lower when cells were transfected with both miR-425 and miR-155 (0.64±0.04 vs. 1.00±0.02, *P*<0.0001) (Fig 2). Combining miR-425 and miR-425 resulted in a significantly greater decrease in *NPPA* expression than did either miR-425 (*P*<0.0001) or miR-155 alone (*P*<0.0001).

**Fig 3:**
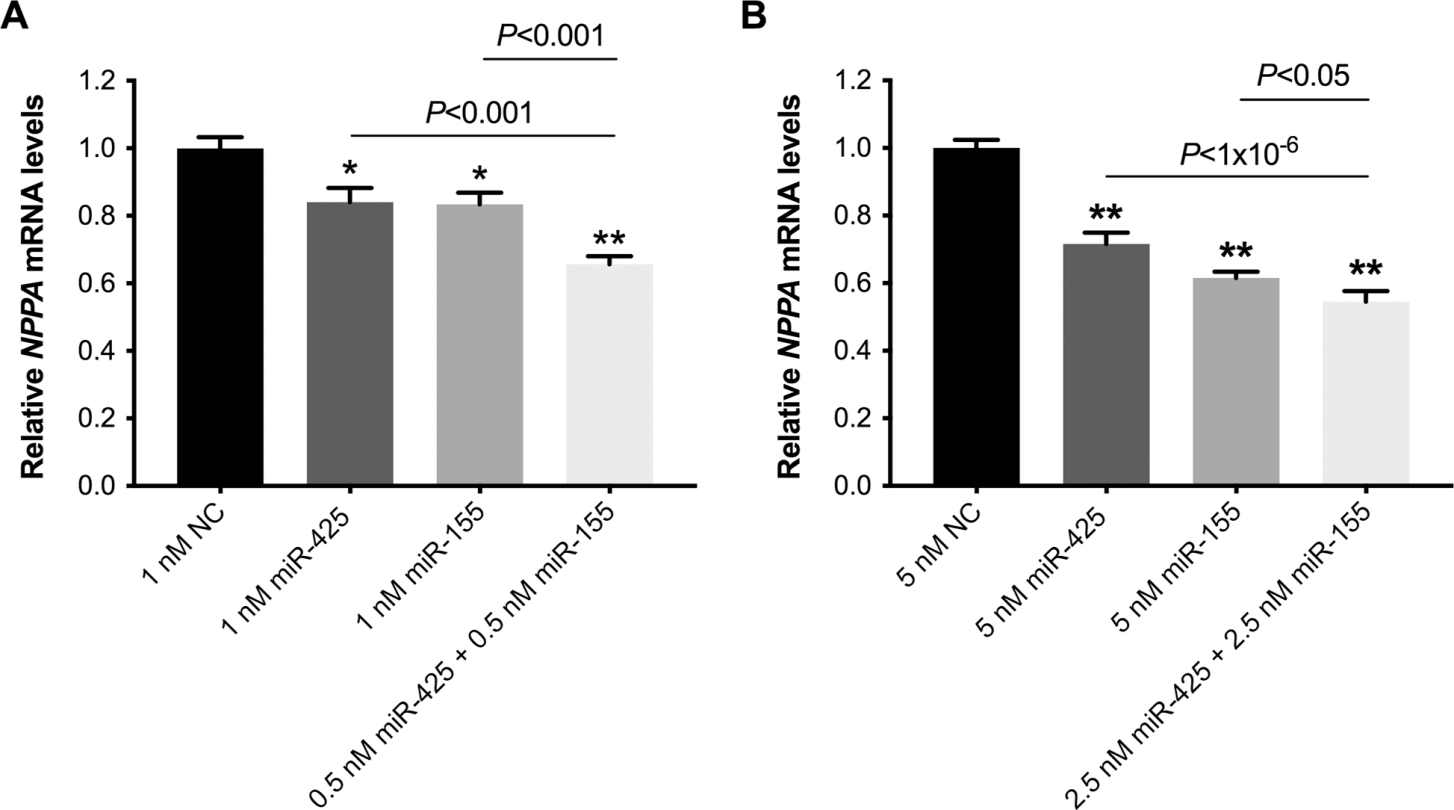
The combination of miR-425 and miR-155 resulted in greater repression of *NPPA* expression than a higher concentration of either miRNA alone. Cardiomyocytes were transfected with 1 nM (A) or 5 nM (B) of miR-425, miR-155, or both miR-425 and miR-155, each at half the concentration of either miRNA alone. Two days later, *NPPA* mRNA levels were measured. *NPPA* expression was normalized to *GAPDH* expression, and shown relative to expression in cells transfected with the negative control miRNA. *P<0.01 and **P<1x10^-6^ versus cells transfected with negative control miRNA. N = 4 experiments (4–12 replicate wells per condition) for (A) and n = 8 experiments (4–12 replicate wells per condition) for (B).

To investigate the effect of combining smaller amounts of each of the two miRs (miR-425 and miR-155) compared to that of each individual miRNA at a higher concentration, *NPPA* mRNA levels were measured in hESC-CMs with 1 nM of miR-425, 1 nM of miR-155, or a combination of 0.5 nM of miR-425 and 0.5 nM of miR-155. Relative to cells transfected with negative control miRNA, *NPPA* expression was 16% lower in cells transfected with miR-425 alone (0.84±0.05 vs. 1.00±0.03, *P* = 0.001), 17% lower when transfected with miR-155 alone (0.83±0.05 vs. 1.00±0.03, *P* = 0.0004), and 34% lower when cells were transfected with the combination of miR-425 and miR-155 (0.66±0.05 vs. 1.00±0.03, *P*<0.0001; Fig 3A). Combining miR-425 and miR-425 resulted in a significantly greater decrease in *NPPA* expression than did either miR-425 (*P* = *P*<0.0001) or miR-155 alone (*P* = 0.0002). Similarly, Nt-proANP protein levels in the cardiomyocyte media were reduced more in cells co-transfected with the two miRNAs (0.52±0.08 vs. 1.00±0.20 in cells transfected with negative control miRNA, *P* = 0.03) than in cells transfected with either miR-425 (0.75±0.06 vs. 1.00±0.20, *P* = 0.26) or miR-155 alone (0.89±0.25 vs. 1.00±0.20, *P* = 0.63; S1 Fig).

The enhanced repressive effect of combining miR-425 and miR-155 on *NPPA* expression was also observed at higher miRNA concentrations. Relative to cells transfected with 5 nM of negative control miRNA, *NPPA* expression was 28% lower in cells transfected with 5 nM of miR425 alone (0.72±0.03 vs. 1.00±0.02, *P*<0.0001), 38% lower when transfected with 5 nM of miR-155 alone (0.62±0.03 vs. 1.00±0.02, *P*<0.0001), and 45% lower in cells transfected with both 2.5 nM of miR-425 and 2.5 nM of miR-155 (0.55±0.03 vs. 1.00±0.02, *P*<0.0001; Fig 3B). The combination of miR-425 and miR-425 resulted in a significantly greater decrease in *NPPA* expression than did either miR-425 (*P*<0.0001) or miR-155 alone (*P* = 0.04).

Taken together, these results demonstrate that *NPPA* repression is greater when administering lower concentrations of miR-425 and miR-155 combined than when administering higher concentrations of either miR-425 or miR-155 alone.

### The combination of miR-425 and miR-155 results in an additive (inhibitory) effect on cGMP levels

**Fig 4:**
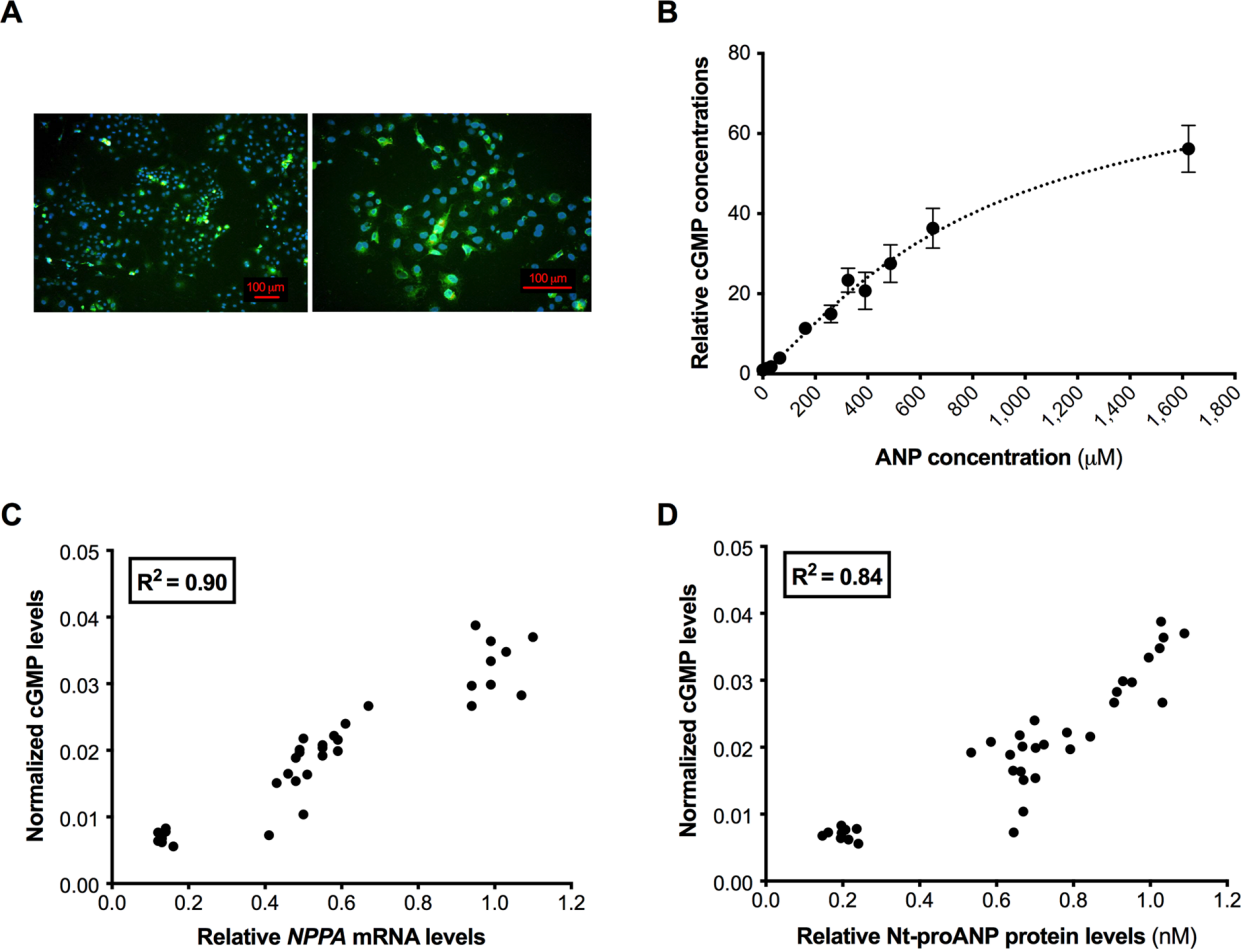
Development and validation of a biological assay to measure the effects of miRNA-mediated changes in *NPPA* expression on cGMP levels. TurboGFP (tGFP) fluorescence in COS7 cells 48 hours after transfection with the NPR1-tGFP expression vector (A). Cell nuclei were visualized using DAPI. The effect of incubating NPR1-expressing COS7 cells with increasing amounts of human ANP on cGMP concentrations (B). Cyclic GMP levels were expressed as picomoles of cGMP per mg of protein, relative to the cGMP levels at baseline (cells not exposed to ANP). The correlation between cGMP levels in NPR1-expressing COS7 cells exposed to media from cardiomyocytes transfected with miRNA(s), and cardiomyocyte *NPPA* mRNA levels (C) or Nt-proANP levels in the cardiomyocyte media (D).

A biological assay was developed to assess the effect of modulating *NPPA* expression on downstream cGMP levels. To do so, COS7 cells were transfected with DNA encoding the ANP receptor NPR1 fused to tGFP. Immunocytochemistry showed that the distribution of NPR1-tGFP in transfected cells (Fig 4A) was similar to that described by previous investigators [15–17]. When the NPR1-tGFP-expressing COS7 cells were exposed to increasing concentrations of human ANP, a dose-response effect of ANP on cGMP levels was observed (Fig 4B). To investigate the relationship between *NPPA* mRNA, Nt-proANP, and production of cGMP, NPR1-tGFP-expressing COS7 cells were exposed to media collected from hESC-CMs transfected with miR-425 and/or miR-155. The cGMP level in these NPR1-tGFP-expressing cells was highly correlated with *NPPA* mRNA levels (r^2^ = 0.90, Fig 4C) and Nt-proANP protein levels (r^2^ = 0.84, Fig 4D) in the cardiomyocytes. These results indicate that this *in vitro* assay represents a reliable tool to investigate downstream biological implications of modulating *NPPA* expression.

**Fig 5:**
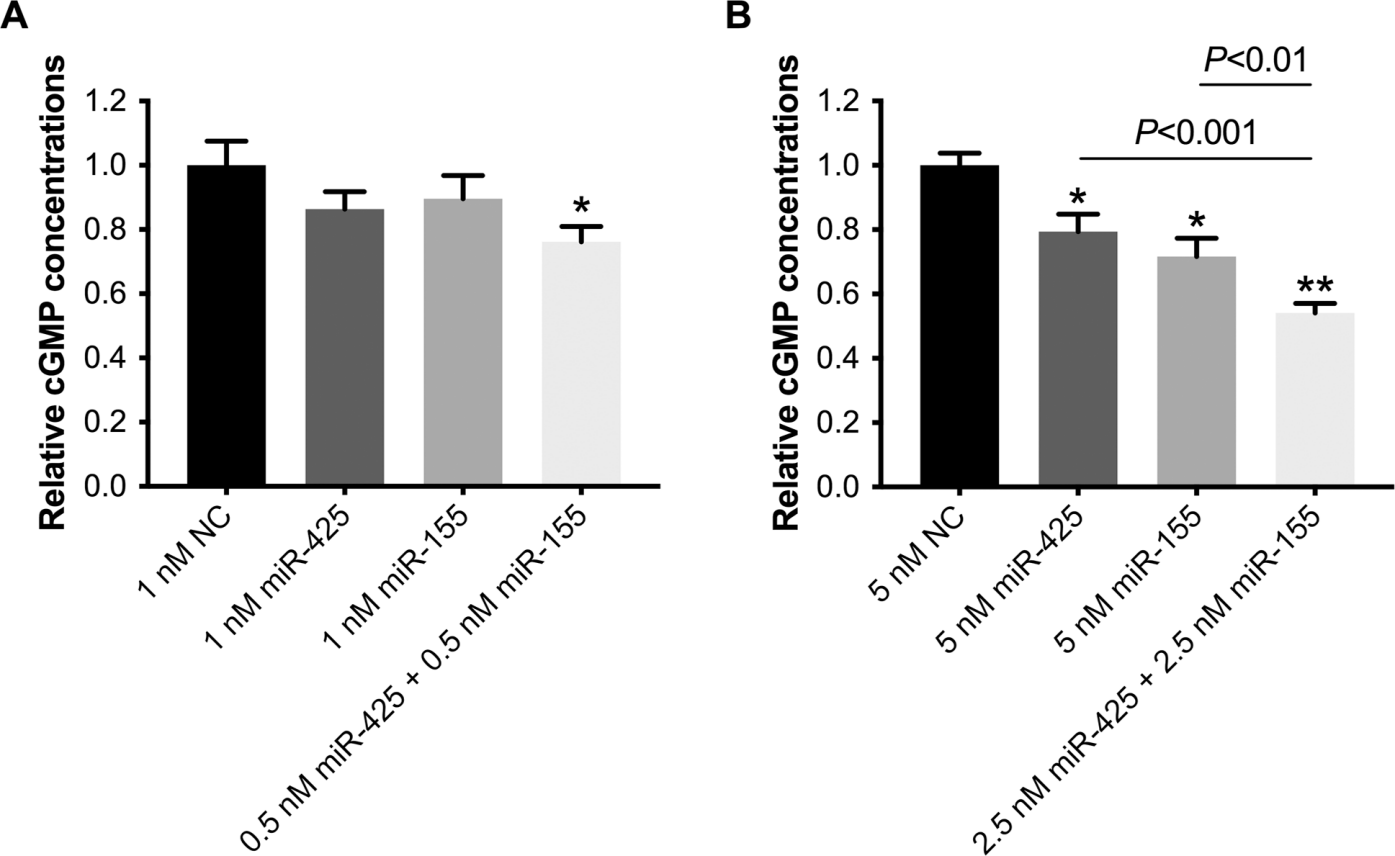
MiR-425 and miR-155 produced an additive decrease in cGMP levels relative to either miRNA alone. Cardiomyocytes were transfected with 1 nM (A) or 5 nM (B) of negative control miRNA (NC), miR-425, miR-155, or the combination of miR-425 and miR-155, each at half the concentration of the other miRs. Cyclic GMP levels were measured in NPR1-expressing cells that were incubated for 2 hours with media collected from the cardiomyocytes. Cyclic GMP levels were expressed as picomoles of cGMP per mg of protein and were shown relative to levels in cells exposed to media from cardiomyocytes transfected with negative control miRNA. *P<0.01 and **P<1x10^-6^ versus cells transfected with negative control miRNA. N = 3 experiments (4–12 replicate wells for each condition) for (A) and n = 8 experiments (4 replicate wells per condition) for (B).

This *in vitro* assay was used to investigate whether the additive effect of combining miR-425 and miR-155 on cardiomyocyte repression of *NPPA* expression resulted in a greater decrease in cGMP levels compared to either miRNA alone. Relative to cells transfected with 1 nM of negative control miR, the cGMP level was 14% lower when cardiomyocytes were transfected with 1 nM of miR-425 alone (0.86±0.09 vs. 1.00±0.07, *P* = 0.12), 10% lower when transfected with 1 nM of miR-155 alone (0.90±0.09 vs. 1.00±0.07, *P* = 0.24), and 24% lower when cells were transfected with the combination of 0.5 nM of miR-425 and 0.5 nM of miR-155 (0.76±0.09 vs. 1.00±0.07, *P* = 0.006; Fig 5A). The relative effects on these down-stream effects of altered *NPPA* expression with 1nM concentrations were lower than effects on *NPPA* expression itself and results were non-significant. We therefore examined cGMP levels with 5 nM concentration of miRNAs.

Relative to cells transfected with 5 nM of negative control miR, cGMP levels were 20% lower when cardiomyocytes were transfected with 5 nM of miR-425 alone (0.80±0.06 vs. 1.00±0.04, *P* = 0.001), 28% lower when transfected with 5 nM of miR-155 alone (0.72±0.06 vs. 1.00±0.04, *P*<0.0001), and 46% lower when cells were transfected with the combination of 2.5 nM of miR-425 and 2.5 nM of miR-155 (0.54±0.06 vs. 1.00±0.04, *P*<0.0001; Fig 5B). Combining miR-425 and miR-425 resulted in a significantly greater decrease in cGMP levels than did either miR-425 (*P*<0.0001) or miR-155 alone (*P* = 0.003). These results are a measure of the biological consequences of the additive effects of combining two miRs that target *NPPA*.

## Discussion

This study demonstrates that combining lower concentrations of miR-425 and miR-155 resulted in enhanced cardiomyocyte repression of *NPPA* gene expression in comparison to higher concentrations of either miR-425 or miR-155 alone. Development of an *in vitro* assay permitted assessment of the potential biological significance of miRNA-induced decreases in *NPPA* expression. The results showed that the cooperative inhibitory effect of miR-425 and miR-155 on *NPPA* expression was associated with a significant decrease in cGMP levels.

Previous investigators have shown that combinations of miRNAs targeting distinct 3’UTR binding sites have cooperative repressive effects on gene expression. For example, Lee and colleagues showed that the combination of miR-130a and miR-495 produced a greater decrease in the expression of the tumor suppressor RUNX3, than either miRNA alone [18]. Similarly, Doxakis showed that the combination of miR-7 and miR-153 resulted in a greater reduction in the expression of α-synuclein, the major component of pathological Lewy bodies in the brain, compared to either miRNA alone [19]. In human melanoma cells, miR-93 and miR-572 displayed cooperative inhibitory effects on the expression of cyclin-dependent kinase inhibitor p21 [20]. In this study, we show that miR-425 and miR-155 cooperatively inhibit *NPPA* gene expression to a greater extent than either miRNA alone in hESC-derived cardiomyocytes and that the effect is sufficient to have biologic consequences in terms of decreasing cGMP production.

Coinheritance of rs5068 and rs61764044 minor alleles is associated with lower blood pressure and our prior work has demonstrated that these alleles interrupt the negative regulatory effect of miR-425 and miR-155, respectively [10]. This observation suggests that combining anti-miRs targeting miR-425 and miR-155 could represent an effective therapeutic approach to treat hypertension. Several phase I and II trials investigating anti-miRs as a therapy for indications other than cardiovascular disease imply the feasibility and potential of anti-miR therapies. These include anti-miR-21 (RG-012) for treatment of Alport syndrome [21], anti-miR-103/107 (RG-125/AZD4076) for type 2 diabetes [22], and anti-miR-155 (MRG-106) for treatment of patients with certain lymphomas and leukemias [23].

Just as treatment with high miRNA concentrations might be expected to have off-target effects (miRNAs target multiple genes), we anticipate that (near) complete depletion of a miRNA (as might occur with high concentrations of an anti-miR) would also have unintended consequences. For example, in addition to decreasing the level of mRNA encoding ANP, miR-155 plays a crucial role in inflammation, immune response, hematopoietic lineage differentiation, tumorigenesis, and cardiovascular diseases [23–30]. Complete or near complete depletion of miR-155 would be expected to affect these other processes. In fact, Rodriguez and colleagues generated a knock-out mouse that lacks miR-155 [29]. These mice are immunodeficient and display increased lung airway remodeling [29]. We anticipate that the combination of submaximal concentrations of two or more anti-miRs (for example, anti-miR-425 and anti-miR-155) may specifically produce the desired effect (increased *NPPA* mRNA) without significantly altering other pathways that are regulated by either miR-425 or miR-155. This hypothesis remains to be tested.

In summary, our study demonstrates that miR-425 and miR-155 cooperatively regulate *NPPA* expression in human cardiomyocytes. The changes in *NPPA* mRNA and ANP levels resulted in changes in cGMP that could be detected using a biological assay. These observations provide the basis for future studies examining whether inhibiting cooperative miRNAs targeting *NPPA* expression could represent an effective strategy to increase ANP levels and thus treat hypertension.

## Supporting information

S1 Fig The combination of miR-425 and miR-155 had an additive, negative effect on the production of Nt-proANP. Nt-proANP protein levels in the media of hESC-CM transfected with negative control miRNA (NC), miR-425, miR-155, or with a combination of miR-425 and miR-155. Nt-proANP protein levels were expressed relative to levels in cells transfected with the negative control miRNA. *P<0.05 versus cells transfected with the negative control miRNA. N = 1 experiment (6 replicate wells per condition).
(TIFF)
