# Supplementary figures and images for: MicroRNA-425 and microRNA-155 cooperatively regulate atrial natriuretic peptide expression and cGMP production
Source: PLoS One. 2018 Apr 26;13(4):e0196697. doi: 10.1371/journal.pone.0196697 (PMC5919659; doi:10.1371/journal.pone.0196697)

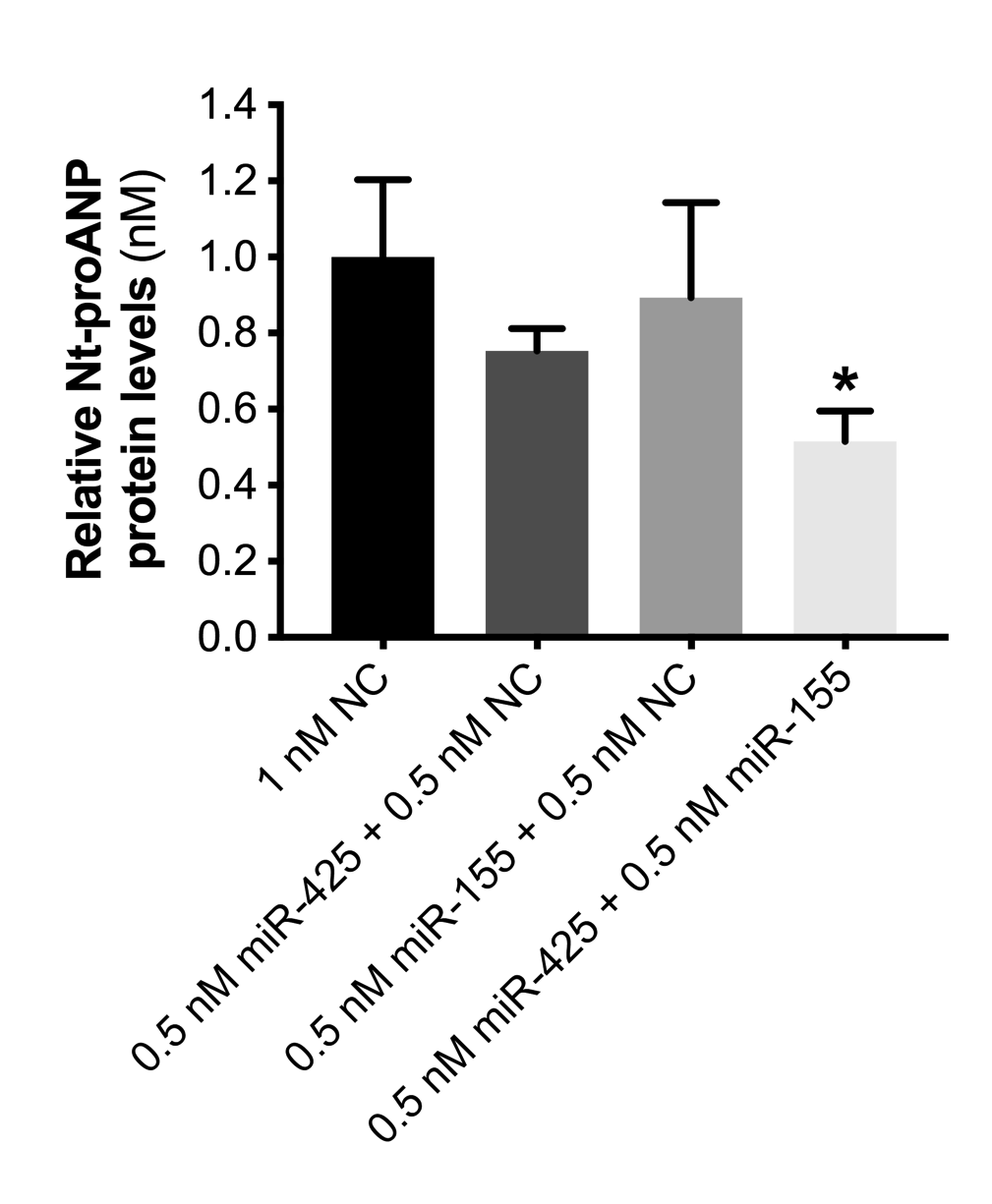

Supplement: S1 Fig — Nt-proANP protein levels in the media of hESC-CM transfected with negative control miRNA (NC), miR-425, miR-155, or with a combination of miR-425 and miR-155. Nt-proANP protein levels were expressed relative to levels in cells transfected with the negative control miRNA. *P<0.05 versus cells transfected with the negative control miRNA. N = 1 experiment (6 replicate wells per condition). (TIFF) [file pone.0196697.s001.tiff]
